# Benefits of Exercise Training for Children and Adolescents Undergoing Cancer Treatment: Results From the Randomized Controlled MUCKI Trial

**DOI:** 10.3389/fped.2020.00243

**Published:** 2020-06-05

**Authors:** Sandra Stössel, Marie A. Neu, Arthur Wingerter, Wilhelm Bloch, Philipp Zimmer, Claudia Paret, Khalifa El Malki, Freerk T. Baumann, Alexandra Russo, Nicole Henninger, Nadine Lehmann, Henrike Otto, Jörg Faber

**Affiliations:** ^1^Center for Pediatric and Adolescent Medicine, Childhood Cancer Center, University Medical Center Mainz, Mainz, Germany; ^2^Department of Molecular and Cellular Sport Medicine, Institute of Cardiovascular Research and Sports Medicine, German Sport University Cologne, Cologne, Germany; ^3^Institute of Sport and Sport Science, Technical University of Dortmund, Dortmund, Germany; ^4^Department I of Internal Medicine, Center of Integrated Oncology Köln Bonn, University Hospital of Cologne, Cologne, Germany

**Keywords:** resistance training, endurance training, physical activity, pediatric oncology, muscle strength

## Abstract

**Objective:** In cancer patients, the impairment in muscle function is a frequently observed phenomenon. However, comprehensive evaluation of the effect of exercise training on muscle function in childhood cancer patients (CCPs) is sparse and therefore investigated in the MUCKI trial.

**Study Design:** In the randomized controlled MUCKI trial, CCPs during intensive cancer treatment and aged 4–18 years were recruited. Eligible patients were enrolled soon after diagnosis as long as they were physically and mentally able to participate in exercise testing and training. Patients of the exercise group (*n* = 16) participated in average 2.7 ± 1.2 times per week in a combined resistance and endurance training with moderate exercise intensity, for a time period of 8.0 ± 2.1 weeks, while patients of the control group (*n* = 17) received usual care. Leg strength was evaluated as the primary endpoint. Secondary endpoints were 6-min walk performance, arm strength, body composition, fatigue, and health-related quality of life.

**Results:** Comparisons of pre- and post-intervention results were evaluated by baseline and stratification criteria adjusted analysis and showed positive effects for the exercise group regarding leg strength [*F*_(1, 20)_ = 5.733; *p* = 0.027^*^; $\eta_{\text{p}}^{2}$ = 0.223], walking performance [*F*_(1, 25)_ = 4.270; *p* = 0.049^*^; $\eta_{\text{p}}^{2}$ = 0.146], fatigue [*F*_(1, 13)_ = 8.353; *p* = 0.013^*^; $\eta_{\text{p}}^{2}$ = 0.391], self-esteem [*F*_(1, 6)_ = 6.823; *p* = 0.040^*^; $\eta_{\text{p}}^{2}$ = 0.532], and self-reported strength and endurance capacity [*F*_(1, 6)_ = 6.273; *p* = 0.046^*^; $\eta_{\text{p}}^{2}$ = 0.511]. No significant differences were found for the other parameters.

**Conclusion:** Within one of the first randomized controlled trials, the present study provides evidence for a positive effect of combined training in CCPs during intensive cancer treatment. Further research is needed to confirm these results and to evaluate their clinical impact.

**Clinical Trial Registration Number:** NCT02612025.

## Introduction

Skeletal muscle dysfunction is a commonly observed phenomenon in cancer patients (1). The disease itself and also its treatment negatively affect skeletal muscle strength and mass and lead to a reduced physical activity (PA) level, which further exacerbates skeletal muscle impairment (2, 3). However, exercise intervention studies in adult cancer patients have shown the effectiveness of physical exercise to increase patients' muscle mass and strength during and after medical treatment. This increase was associated with a significant improvement in patients' quality of life and with a reduction of frequently observed side effects, including reduced whole-body muscle mass and fatigue (4, 5). Strength and endurance training are recommended to generate positive effects on muscle function (3).

In children and adolescents suffering from cancer, lower limb muscle function seems to be more affected than upper limb muscles (6, 7). In children suffering from acute lymphoblastic leukemia, an impairment in leg muscle strength was observed within 7–10 days after diagnosis (8). In survivors of acute lymphoblastic leukemia during childhood, decreased muscle mass as well as impaired leg muscle strength and mobility deficits were revealed, and it is presumed that this may reduce the ability to participate in PA and lead to further fitness deficits (6). For adult cancer patients, exercise guidelines were developed by the American College of Sports Medicine in 2019 (9). For childhood and adolescent cancer patients (CCPs), specific training programs need to be established that are adapted to the CCPs' needs regarding their mental and physical development, the specific entities of childhood cancer and their social environment. Furthermore, the different entities of childhood cancer being associated with specific requirements and restrictions need to be considered, and training conditions need to be adjusted individually. Recent reviews and meta-analysis showed that the level of evidence for exercise training efficiency in CCPs during intensive cancer treatment is low, and further randomized controlled trials to analyze exercise training in pediatric cancer were requested (10–12).

For the MUCKI trial (clinical trial registration number: NCT02612025), a specific and individualized combined strength and endurance exercise program was designed for CCPs. Regarding the above-described muscle function impairments especially in the lower limbs in pediatric cancer patients, the primary outcome of the present study was to investigate whether the adapted exercise program can improve leg muscle strength and other physical and mental parameters (secondary outcomes) in CCPs during intensive cancer treatment.

## Materials and Methods

The MUCKI trial was carried out at the Childhood Cancer Center at the Center for Pediatric and Adolescent Medicine at the University Medical Center Mainz in Germany. All procedures performed in this study are in accordance with the ethical standards of the institutional research committee [approved by ethics review committee of the Rhineland-Palatinate Chamber of Physicians, trial reference number 837.059.15 (9827)] and with the 1964 Declaration of Helsinki and its later amendments. Written informed consent was obtained from the legal guardians in the case of minor participants and from all study participants older than 6 years of age.

### Participants

Patients were eligible if they were at least 4 years of age and treated with chemotherapy and/or radiotherapy for an oncologic disease according to the International Classification of Childhood Cancer at the University Medical Center Mainz in Germany. Patients were excluded in case of functional and/or cognitive limitations which might confine performance during training, orthopedic condition which hinders adequate participation in exercise training, heart failure (NYHA III and IV), partial or global respiratory failure, symptomatic coronary disease, serious therapy-refractory hypertonia, sustainable thrombocytopenia <10,000/μl, hereditary or acquired thrombocytopenia or coagulation disorder, uncontrolled seizure, central nervous system metastases, and any other medical or psychological condition which did in the physician's opinion not allow participation in sport activity or which might limit performance during testing or training.

### Procedure

A treating oncologist enrolled children and adolescents meeting the inclusion and exclusion criteria soon after diagnosis as long as they were physically and mentally able to participate in exercise testing and training. After a pre-intervention exercise test, the study participants were randomly assigned in a 1:1 ratio in either an exercise group (EG) or a control group (CG). Beyond usual medical care, participants from the EG took part in a 6- to 8-week-long supervised exercise program, whereas patients from the CG received usual care without a supervised exercise intervention. A post-intervention exercise test took place for participants of both study groups about 6–8 weeks after pre-intervention testing. All exercise tests were programmed to take place prior to the start of a new cancer therapy cycle on condition of hematologic recovery in order to ensuring maximum comparability.

### Randomization

Stratified randomization was used to equally allocate (1:1) patients in the EG and CG. Following the minimization procedure according to Pocock and Simon (13), tumor entity (acute leukemia or t-cell lymphoma/central nervous system tumor/others), age (at least 12 years of age/younger than 12 years of age), sex, and physical performance status assessed by Lansky score at diagnosis (higher than 50/equal or lower than 50) were used as stratification factors. Computerized randomization software [Randomization In Treatment Arms (RITA) Version 1.31, StatSol Lübeck, Germany] was used for this purpose. The investigator, therapists, and patients were not blinded to group assignment as it was apparent to which patients the exercise intervention was provided.

### Sample Size

Prior to the MUCKI trial, power calculation was not achievable due to the lack of reliable data from previous studies. Based on an interim analysis after inclusion of 20 patients in the MUCKI trial, power calculation indicated that a sample size of 35 patients was needed to determine intergroup differences in leg muscle strength (power set at an 80% level, two-sided two-sample *t*-test set at a 0.05 significant level). The software G^*^Power 3.1 was used for this calculation.

### Study Parameters and Methods

All of the following described measurements were undertaken at pretesting and posttesting except the PA questionnaire, which was solely filled out at the pretest. Contraindications for exercise testing and training were fever/body temperature of >38.0°C; acute infection/sepsis; severe pain; acute bleeding; thrombocytes <20/nl (thrombocytes <50/nl no strength testing); nausea/vomiting/diarrhea; resting heart rate >130/min (<12 years of age) and >100/min (≥12 years of age); cardiovascular disorders; ongoing mediastinal irradiation; vertigo; severe ataxia; disturbance of consciousness; other medical or psychological conditions denying medical clearance by the treating oncologist; and patient or parent denying participation.

#### Anthropometric Data and Medical Data

Anthropometric data on body height and weight were measured using standardized methods and equipment. Medical data, such as information on diagnosis and treatment, were obtained from the patient records.

#### Muscle Strength

Knee strength and elbow flexor strength were measured with a handheld dynamometer (CITEC handheld dynamometer, type 3002, CIT Technics, Haren, Netherlands). Maximum isometric contraction was evaluated using the “break technique” described by Beenakker et al. (14). The highest value out of three repetitions was counted as the maximum strength and kept for analysis. The handheld dynamometry method showed high validity (*r* = 0.74 and *r* = 0.98) and moderate intertester reliability (*r* = 0.42 to *r* = 0.73) in healthy and chronically ill children and adolescents (15, 16) and is considered as a reliable and valid instrument for muscle strength assessment in a clinical setting compared to the gold standard isokinetic dynamometry (17). In the MUCKI trial, only two exercise practitioners performed the strength test throughout the entire study time to decrease interrater variability. The minimally important difference of this measurement is not known for the healthy population or for cancer patients. However, Vaidya et al. (18) described a minimally important difference of 7.5 Newton for fixed dynamometer testing in patients with chronic obstructive pulmonary disease.

#### Walking Performance

To evaluate walking performance, the 6-min walk test (6 MWT) was performed according to the test protocol from Geiger et al. (19) for 6 MWT in children. The 6 MWT was conducted on a 15-m-long course in a hospital corridor. The test showed moderate validity (*r* = 0.44 and *r* = 0.76) and high reliability in healthy and chronically ill children and adolescents (*r* = 0.90 und *r* = 0.94) (20, 21).

#### Body Composition

Bioelectrical impedance analysis (BIA EgoFit, 2010, Germany) was used to measure phase angle (phA). The phA is a well-established indicator for cellular health where higher phA values signify better quality of soft tissue (22). In healthy adults and cancer patients, the phA correlated to overall mortality, clinical outcome, PA level and exercise performance (22). The validity of phA measurement was evaluated in healthy normal-weight and underweight children, where phA was correlated to body weight and arm circumference (*r* = 0.818 and *r* = 0.901) (23). Measurements with the BIA method at 50 kHz are known for high reliability (age-dependent *p* = 0.003 to 0.001) (24) and were performed in the MUCKI trial on the right body side for the whole body, arm, and leg evaluations.

#### Fatigue

To evaluate fatigue level, the German language questionnaire PedsQL 3.0 Multidimensional Fatigue Scale (25) was used. It consists of 18 items about general fatigue, sleep/rest fatigue, and cognitive fatigue. Both child and parent proxy questionnaires were filled out.

#### Health-Related Quality of Life (HRQOL)

The German-language KINDL questionnaire (26) was filled out by the study participants to evaluate HRQOL. It contains 30 items concerning physical and mental well-being, self-esteem, family, friends, and functionality in daily life. In addition, cancer-specific questions about the disease and treatment were posed. Both child and parent proxy questionnaires were filled out.

#### PA Questionnaire

The level of PA prior to disease onset (before any signs or symptoms of the disease were present) was determined by the German MoMo questionnaire (27). The questionnaire consists of items asking for PA level and intensity in daily life, at kindergarten/school, and in sports clubs. The information provided by the questionnaire was used to adapt the training program to individualize the exercise training. Adolescents completed the questionnaire on their own, whereas parents helped younger children to fill it out.

#### Semi-Structured Interview

In a semi-structured interview, patients' PA behavior was evaluated. For this, patients rated their PA level on a 10-stage scale and that for children under the age of 8 years on a three-stage scale, with 1 meaning no PA and 10 signifying a very high PA level. Further, patients were asked how many hours they spent out of bed during (a) homestays and (b) hospitalization (28). Barriers and motivation factors for PA were evaluated by open questions. Patients from the EG were asked at the post-intervention test to evaluate the exercise program in which they had participated during the trial.

#### Intervention

Patients from the EG participated in a supervised exercise training for 6–8 weeks during intensive cancer treatment. The training took place in the inpatient and outpatient clinics as well as in patients' home during outpatient stays. Most of the exercise sessions were supervised by an exercise scientist. Occasionally, such as on weekends, patients and their parents trained on their own following exercise recommendations from the exercise scientist.

The aim was to accomplish three weekly training sessions of 45–60 min each. However, training workload was adapted to the patient's exercise habits and fitness and health condition. Training mainly consisted of age-appropriate and moderate-intensity endurance and strength training. Additionally, active games and balance and stretching exercises were performed. Endurance training comprised mainly ergometer training, aerobic exercises, and walking exercises. Strength training was mostly performed on a cable pulley machine, with mini dumbbells, elastic bands, or body weight. The exercises were surrounded by playful games in order to enhance compliance especially for younger children. Compliance for training was rated by the supervising exercise practitioner at the end of each session on a 6-point scale with 1 presenting the highest compliance.

A training session started with playful exercises such as low-intensity ballgames, followed by moderate-intensity endurance and muscle strength exercises which were often combined with coordination and balance exercises. A cooldown at the end of each session consisted of light-intensity playful exercise, relaxation, and stretching.

Exercise intensity was rated as moderate if at least two of the following criteria were fulfilled: (1) 60–75% of estimated maximum heart rate (29), (2) a score of 12 to 13 on the Borg scale RPE6-20 (30), of 11–12 for playful activities such as exergaming (31), and at medium level on a child-adapted three-level scale (32), (3) strength exercises were rated as moderately intense if no more than two to three more repetitions could be realized from the patient with correct technical execution (progression to no more possible further repetition was aimed over the weeks of training) (33, 34), and (4) a rating of the intensity level by the supervising exercise scientist depending on breathing rate, sweating, and other signs of exhaustion. The goal was to perform 15–20 min of moderate endurance training per session as well as 6–10 min of leg strength training. For homestays, the participants and parents of younger children were trained to use the Borg scale and to use a provided wrist-worn heart rate monitor (Polar A360, Electro, Kempele, Finland) to control the exercise intensity. Additionally, heart rate was collected manually to detect eventual measuring errors of the wrist-worn heart rate monitor (35).

A 20-m^2^-sized exercise room was available for training in the children's hospital. Exercise sessions were conducted with a single patient or a patient group and could be accompanied by parents, siblings, or friends.

The equipment used for training met hygienic requirements needed in the oncological setting, was transportable to the patient's room, and was available for different body sizes. Medical clearance to participate in exercise was obtained prior to any exercise test or session.

### Statistical Methods

Group–time interaction was evaluated by analysis of covariance (ANCOVA) adjusted for the baseline value and stratification criteria (i.e., age, sex, tumor entity, and Lansky score at diagnosis). Results from ANCOVA are reported as *p*- and *F*-values, degrees of freedom (df), and effect size (partial η^2^). Pearson *r* was used to evaluate correlations. The level of significance was set at *p* ≤ 0.05. Statistical analysis was performed using IBM SPSS Statistics software Version 23. All available data were included in the statistical analysis referring to the intention-to-treat approach with respect to the original study group assignment and regardless of the patient's participation rate in the exercise intervention. Only completed outcome measures were included in the analysis, and no attempt to impute missing data was made.

## Results

**Figure 1:**
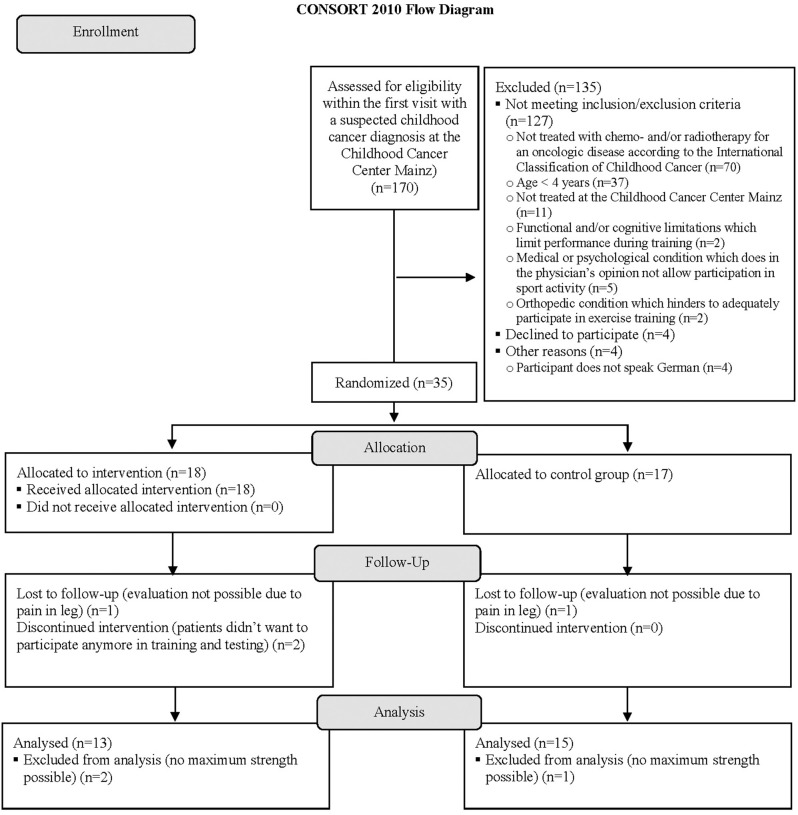
Flow diagram of the trial progress. Adapted CONSORT 2010 flow diagram (36) of the progress through the phases of the randomized controlled MUCKI trial of the exercise group and control group regarding the primary outcome knee flexor strength.

Enrollment of study patients took place from December 2015 to September 2018. After screening for inclusion and exclusion criteria, 18 patients were randomized to the EG and 17 to the CG. In the EG, there was one dropout due to lack of motivation for participation in exercise training and another dropout due to unstable mental condition prohibiting participation in regular exercise training and testing. The primary outcome could not be determined in four participants due to leg pain and in another participant because of a technical problem with the dynamometer. Additional information concerning the progress of all participants during the trial is shown in the flow diagram (Figure 1) adapted from the CONSORT 2010 Statement (36). Patient characteristics are presented in Table 1. At baseline, patients achieved 53.25 ± 33.21% of leg muscle strength and 62.30 ± 14.31% of arm muscle strength compared to norm values (14). Results from BIA revealed significantly lower phA in patients compared to norm values (37) (*p* = 0.000^*^; *d* = 1.05).

**Table 1:** Characteristics of the study population.

	**Exercise group (*n* = 16)**	**Control group (*n* = 17)**	***p*-value**
Age (year)	10.6 ± 5.19 (4.1–17.3)	11.4 ± 4.25 (5.1–17.7)	0.476
Sex (*n* boys)	10	10	–
Body height (cm)	144.4 ± 27.5	146.0 ± 20.5	0.849
Body weight (kg)	42.3 ± 19.8	44.1 ± 25.4	0.961
BMI (kg/m^2^)	19.1 ± 3.5	19.2 ± 5.7	0.831
BMI (*z*-score)	−0.07 ± 0.75	0.07 ± 1.22	0.704
Lansky score at diagnosis < 50 (*n*)	2	1	–
Tumor entities (*n*)			–
-Leukemia/T-cell-lymphoma	8	7	
-CNS-tumor	2	2	
-Others	6	8	
Time from pretest to posttest (week)	8.0 ± 2.1 (5.1–12.1)	8.4 ± 2.6 (5.4–14)	0.687
Time from diagnosis to pretest (week)	7.3 ± 3.7	5.9 ± 3.0	0.354
PA level from the MoMo questionnaire	89.0 ± 90.9% of norm	101.4 ± 49.0% of norm	0.628

*Data are mean ± SD, n or range; CNS, central nervous system; PA, physical activity; data of the two dropped-out participants are not included in the table*.

Comparing intergroup baseline results after the adjustment for the stratification criteria, only leg muscle strength was significantly different between the two groups with lower strength in the EG (*p* = 0.002^*^; *d* = 0.378).

On average, each patient performed 2.7 ± 1.2 supervised exercise sessions per week during an 8.0 ± 2.1-week-long intervention period. One session lasted 48.1 ± 10.1 min, from which most of the time was spent for endurance (18.1 ± 9.1 min in total including 10.4 ± 4.5 min of moderate intensity) and leg strength exercises (16.7 ± 8.4 min in total including 10.5 ± 3.3 min of moderate intensity). Most training sessions were supervised, and few sessions were self-administered (Table 2). Compliance for training was rated 1.7 ± 0.5 on an above-explained 6-point scale. Exercise scientists supervised 85.1 ± 16.7% of all sessions performed, and the remaining sessions were conducted by the patients on their own. The leading cause of cancelation or postponing of exercise sessions were medical reasons, representing 53.7% (i.e., infections/cough/dental inflammation 13.6%; reduced platelet count requiring transfusion 12.2%; severe nausea/vomiting 6.8%; fatigue/exhaustion 6.8%; severe bone pain 2.7%; others 7.5%). Other reasons were lack of motivation for autonomous training not being supervised by an exercise scientist (15.0%), lack of time/staff/room (14.3%), lack of motivation for supervised training (4.8%), parents refusing their child's participation in an exercise session (2.0%), and other reasons (10.2%).

**Table 2:** Overview of training settings in the MUCKI trial.

**Setting of the training session**	**Number of sessions in total (*n*)**
Inpatient setting	9.3 ± 3.9
Outpatient setting	2.1 ± 1.4
Supervised training in patient's home	4.9 ± 4.9
Self-administered training	3.4 ± 5.3

*Results as means ± SD*.

Regarding muscle strength, ANCOVA revealed benefits for knee flexor strength in the EG [*F*_(1, 20)_ = 5.733; *p* = 0.027^*^; $\eta_{\text{p}}^{2}$ = 0.223] with no differences in arm flexor strength [*F*_(1, 21)_ = 1.108; *p* = 0.305] (Table 3). Additionally, walking performance investigated by the 6 MWT showed positive group–time interaction effects for the EG [*F*_(1, 25)_ = 4.270; *p* = 0.049^*^; $\eta_{\text{p}}^{2}$ = 0.146] (Table 3). Neither at the pretest nor at the posttest an intergroup difference was observed in maximally achieved heart rate or rate of perceived exertion (RPE). The heart rate in percent of age predicted maximum heart rates of 79.5 ± 9.7 % in the EG and 79.6 ± 13.8% in the CG (*p* = 0.971) at the pretest and of 82.9 ± 9.0% in the EG and 76.2 ± 11.0% in the CG (*p* = 0.156) at the posttest. The RPEs were 9.6 ± 3.9 in the EG and 10.9 ± 2.6 in the CG (*p* = 0.415) at the pretest and 11.5 ± 2.7 in the EG and 8.6 ± 5.7 in the CG (*p* = 0.259) at the post-test. As mentioned in the Materials and Methods section, questionnaires for patient's fatigue and HRQOL were filled out by patients and their parents separately. Reasons for failed questionnaire completion were low compliance, lack of time, insufficient German speaking ability, and the absence of patient's parents on the examination day (Table 3). The patients' self-reported fatigue level was significantly decreased at the time of the posttest relative to the pretest in EG (*p* = 0.026^*^; *d* = 1.11), but not in CG. However, results from ANCOVA did not reveal significant differences in group–time interaction for the self-reported fatigue level. Patients' fatigue levels reported by their parents illustrate a positive effect of the exercise training [*F*_(1, 13)_ = 8.353; *p* = 0.013^*^; $\eta_{\text{p}}^{2}$ = 0.391]. HRQOL evaluated by the patients themselves suggested beneficial effects in the EG regarding the subscales self-esteem [*F*_(1, 6)_ = 6.823; *p* = 0.040^*^; $\eta_{\text{p}}^{2}$ = 0.532] and strength and endurance [*F*_(1, 6)_ = 6.273; *p* = 0.046^*^; $\eta_{\text{p}}^{2}$ = 0.511]. No significant group–time interactions were seen in patients' HRQOL reported by their parents. Moreover, no significant group–time interactions were found for the phA measured by BIA (Table 3). In the interview, patients from EG indicated higher PA levels than those from CG for the group–time interaction [*F*_(1, 20)_ = 7.255; *p* = 0.014^*^; $\eta_{\text{p}}^{2}$ = 0.266], whereas no significant difference was reported regarding the time spent out of bed in the clinic as well as at home during homestays. Moreover, all patients of the EG reported that they had fun taking part in the exercise program and that they would recommend it to other patients. Furthermore, all of them would appreciate continuing to take part in the exercise offers during hospital stays.

**Table 3:** Results from pretests and posttests by group.

	**Exercise group**			**Control group**			**Interaction**
	**Pretest**	**Posttest**	***n***	**Pretest**	**Posttest**	***n***	***p*-value**
**Muscle strength**							
Knee flexor (*N*)	74.62 ± 41.45	89.62 ± 45.99	13	128.13 ± 49.78	115.60 ± 44.48	15	^*^0.027
Arm flexor (*N*)	92.36 ± 41.18	93.86 ± 42.39	14	103.80 ± 45.27	95.53 ± 37.49	15	0.305
**Walking performance**							
6 MWT (*m*)	470.66 ± 179.75	515.29 ± 136.18	16	531.82 ± 81.52	517.32 ± 54.53	17	^*^0.049
**Anthropometry**							
phA whole body (Ω)	4.63 ± 0.97	4.61 ± 0.78	12	5.12 ± 0.68	5.00 ± 0.82	17	0.180
phA leg (Ω)	3.86 ± 1.32	3.79 ± 1.07	12	4.44 ± 1.08	4.21 ± 1.07	17	0.210
phA arm (Ω)	4.25 ± 0.65	4.12 ± 0.56	12	4.65 ± 0.60	4.61 ± 0.70	17	0.158
Body mass (kg)	39.33 ± 18.89	40.91 ± 21.65	13	44.07 ± 25.44	44.12 ± 24.95	17	0.149
BMI (kg/m^2^)	18.63 ± 3.47	19.02 ± 4.59	13	19.23 ± 5.70	19.24 ± 5.80	17	0.416
**Fatigue (total score)**							
Patient questionnaire	70.55 ± 17.43	79.17 ± 12.00	7	71.18 ± 18.14	70.96 ± 21.69	11	0.327
Parent questionnaire	63.00 ± 21.98	77.19 ± 13.40	9	61.57 ± 18.87	68.34 ± 8.65	12	^*^0.013
**HRQOL (total score)**							
Patient questionnaire	63.78 ± 9.16	68.75 ± 9.46	5	81.11 ± 12.32	77.73 ± 14.70	9	0.504
Parent questionnaire	71.31 ± 9.07	71.03 ± 11.80	7	73.00 ± 13.02	70.53 ± 12.99	14	^*^0.046
**Interview**							
PA level (scores 1–10)	5.00 ± 3.16	6.89 ± 1.80	13	6.00 ± 3.02	4.87 ± 3.21	15	^*^0.014
Hours out of bed in clinic	4.14 ± 3.95	2.64 ± 1.60	7	5.98 ± 6.09	5.14 ± 5.58	9	0.445
Hours out of bed at home	9.36 ± 4.53	13.29 ± 1.98	7	7.17 ± 3.95	8.47 ± 5.81	9	0.099

* *p ≤ 0.05; data are mean ± SD; phA, phase angle; HRQOL, health-related quality of life; PA, physical activity*.

Analysis revealed significant correlations between before and after changes from 6 MWT and (a) three subscales from patients' self-reported HRQOL (i.e., physical well-being *r* = 0.751; *p* = 0.003; mental well-being *r* = 0.633; *p* = 0.020; self-esteem *r* = 0.597; *p* = 0.024), (b) patients' self-esteem reported by their parents (*r* = 0.442; *p* = 0.035), (c) patients' fatigue reported by their parents (*r* = 0.528; *p* = 0.014). Regarding the other subscales of fatigue and HRQOL questionnaires, no correlations with the walking performance were found. Furthermore, no correlations were observed concerning before–after changes in leg strength and fatigue or HRQOL.

### Adverse Events

No serious adverse events occurred during the, in total, 381 exercise and testing sessions. During walking exercises in testing and training sessions, three patients fell but without getting injured. Furthermore, light to moderate muscle soreness was experienced three times after testing sessions and four times after training.

## Discussion

The MUCKI trial aimed to gain further knowledge about possible effects of exercise training in pediatric oncology, as the level of evidence in this field is rather low. Results from the MUCKI trial showed that combined resistance and endurance training adapted to the specific requirements of pediatric oncology leads to multiple benefits during intensive cancer treatment in a non-stem-cell transplant setting.

The present results revealed positive effects of exercise training on leg muscle strength, but not on arm muscle strength. This, however, can be explained by the fact that the resistance training performed during the MUCKI trial was focused on strengthening the leg muscles, so that an improvement in arm muscle strength could not be expected. Thus, the data from the MUCKI trial confirm that specific leg strength training leads to benefits in the trained muscles in CCPs. The results are in accordance with the study of Fiuza-Luces et al. (38), who observed positive effects on muscle strength following leg and arm training in pediatric patients during neoadjuvant treatment for solid tumors. Consequently, the results from the MUCKI trial indicate that benefits from specific training can also be achieved in pediatric patients with tumor entities other than solid tumors. Higher muscle strength was shown being related to an improved treatment tolerance in adult cancer patients (5) as well as to an increased quality of life in healthy children and adolescents (33). Hence, the present results in CCPs provide evidence for possible positive effects of specific training on muscle strength assuming benefits regarding patient's quality of life. Test failures in the MUCKI trial were mainly caused by leg pain. Future trials should consider failure reasons to increase the participation rate in muscle strength testing.

The MUCKI trial revealed that patients participating in exercise training improve their walking performance, which was accompanied by decreased fatigue and improved HRQOL. Substantiating the results of the present study, a positive association between walking performance and fatigue in pediatric cancer patients during intensive treatment was already observed by Braam et al. (39). Furthermore, better walking performance was associated with greater physical well-being in patients suffering from chronic obstructive pulmonary disease (40). The underlying mechanisms which are responsible for the benefits of exercise on fatigue and well-being are not completely understood to date. It is assumed that there might be direct mechanisms such as enhanced functional capacity and decreased inflammation, as well as indirect mechanisms such as improved sleep quality and decreased anxiety (41). In recent reviews and meta-analyses, it remains unclear whether training benefits on walking performance can be achieved in CCPs (11, 12). The present findings aim to increase the actual low level of evidence in this field. According to the present findings of the MUCKI trial, training of walking capacity seems to be worthwhile in order to attain benefits on walking performance, which come along with positive effects on fatigue and HRQOL. Further research into the underlying mechanisms is required.

In the present population, the phA derived from BIA was significantly lower compared to norm values. Reduced phA was shown being related to lower cellular health and higher morbidity and mortality (22, 42). In previous studies, phA was increased after an exercise training lasting 2–6 months in the elderly and in patients suffering from chronic obstructive pulmonary disease (43–46). However, no significant effect in group–time interaction regarding phA was found in the MUCKI trial. This may be due to measuring bias related to the BIA method, due to varying hydration status in cancer patients (47, 48), to the relatively short time of exercise intervention, or to an incapacity of CCPs to adapt to training on the cellular health level. Further research on potential benefits of training on body composition is of particular interest for CCPs, especially in view of attenuating the cachexia syndrome which is observed in 7–50% of CCPs (49, 50).

Questionnaires investigating the level of fatigue and HRQOL in the MUCKI trial showed beneficial effects for EG in some of the total and sub-scores of the questionnaires. These results are in accordance with findings revealing benefits on fatigue and HRQOL in adult cancer patients (51, 52). The present results confirm the findings of Lam et al. (53) who observed benefits on fatigue and HRQOL after an exercise intervention lasting 6 months during childhood cancer treatment. As in CCPs, the evidence level in this field is low (10, 11) the present results provide important evidence for positive effects of exercise training on fatigue and HRQOL in CCPs. This is of particular interest, as no gold-standard treatment options are established so far to attenuate fatigue (54). However, the questionnaire response rate in the MUCKI trial was low due to the above-explained reasons. In future trials, higher sample sizes should therefore be considered, and additional time for the effortful questionnaire completion should be scheduled besides the PA testing sessions. In the present study, patients indicated that they had fun participating in the exercise training. Furthermore, compliance for the exercise training was high, and no serious adverse events did occur, indicating that the training of the present study was well accepted and tolerated by CCPs. One limitation of the MUCKI trial is the high variance of the data, which is attributed to the high heterogeneity of the study population regarding, for example, a wide range of age, tumor entities, and different medical treatment protocols. Nevertheless, this heterogenic cohort represents the population exercise professionals are confronted to in their daily practice in pediatric oncology.

Another potential limitation is the restricted interpretation of the training effect on leg muscle strength in the MUCKI trial because of the baseline difference in leg muscle strength between the EG and the CG. This may lead to an overestimation of the training effect (55). Further investigations using higher sample sizes are needed to perform subgroup analysis of more homogeneous populations. This would provide a deeper insight into training adaptation and thus allow further optimization of training efficiency in CCPs.

The interpretation of the results from the MUCKI trial is further limited by the small and heterogeneous sample. Although a balanced allocation in both study groups with regard to the stratification factors was aimed within the MUCKI trial, the results need to be confirmed in higher sample sizes, allowing subgroup analysis to evaluate, for example, if different cancer treatments have impacts on the exercise efficiency. However, the heterogeneous sample allows us to put the experiences made in the MUCKI trial into practice as exercise professionals in pediatric oncology are confronted to such heterogeneous populations.

In the MUCKI trial, benefits on leg muscle strength during intensive cancer treatment were observed. Follow-up was not part of the study protocol. Impairments in leg muscle strength were detected during childhood cancer treatment, as well as in survivors after the treatment, as explained in the Introduction. Therefore, potential long-term effects of exercise training on muscle function in CCPs should be investigated in the future.

### Perspective

The MUCKI trial was designed to gain new knowledge about adapted physical exercise in CCPs that might be considered to improve integrated health care in pediatric oncology. Within one of the first randomized controlled trials, this study illustrates the positive effects of exercise training on leg muscle strength in CCPs during intensive cancer treatment. The present findings can be used by exercise professionals to optimize the exercise training with CCPs. As adapted exercise provision is still rare in multidisciplinary pediatric cancer care and specialized staff and facilities to provide adapted exercise are often missing, these new data encourage further investigations on exercise training in pediatric oncology. In this perspective, the confirmation of the present results and the evaluation of their clinical impact are needed.

## Data Availability Statement

The datasets generated for this study are available on request to the corresponding author.

## Ethics Statement

The study was approved by the ethics review committee of the Rhineland-Palatinate Chamber of Physicians, trial reference number 837.059.15 (9827). Written informed consent to participate in this study was provided by the participants' legal guardian/next of kin. Written informed consent was obtained from the legal guardians in the case of minor participants and from all study participants older than 6 years of age.

## Author Contributions

SS, MN, AW, WB, CP, KM, AR, NH, FB, PZ, and JF contributed to the conception and design of the study and contributed to the application for approval at the ethic committee. SS, MN, AW, WB, CP, KM, AR, NH, HO, FB, PZ, and JF analyzed and interpreted the study data. SS and MN drafted the article. AW, WB, CP, KM, AR, NH, NL, HO, FB, PZ, and JF substantially revised the article. SS, MN, AW, KM, AR, NH, and HO acquired the data. SS, MN, AW, KM, AR, NH, and HO recruited the study participants. JF handled funding and supervision.

## Conflict of Interest

The authors declare that the research was conducted in the absence of any commercial or financial relationships that could be construed as a potential conflict of interest.

## Abbreviations

6MWT: 6-min walk test
BIA: bioelectrical impedance analysis
CCPs: childhood and adolescent cancer patients
CG: control group
EG: exercise group
HRQOL: health-related quality of life
PA: physical activity
RPE: rate of perceived exertion.
